# Utility of the clivo-axial angle in assessing brainstem deformity: pilot study and literature review

**DOI:** 10.1007/s10143-017-0830-3

**Published:** 2017-03-03

**Authors:** Fraser C. Henderson, Fraser C. Henderson, William A. Wilson, Alexander S. Mark, Myles Koby

**Affiliations:** 1 Doctor’s Community Hospital, Lanham, MD USA; 2 The Metropolitan Neurosurgery Group, LLC, 8401 Connecticut Avenue, Suite 220, Chevy Chase, MD 20815 USA; 3 Medical University of South Carolina, Charleston, SC USA; 4 University of Maryland Medical Center, Baltimore, MD USA

**Keywords:** Clivo-axial angle, Cervical medullary syndrome, Basilar invagination, Brainstem deformation, Craniocervical instability, Dynamic MRI

## Abstract

There is growing recognition of the kyphotic clivo-axial angle (CXA) as an index of risk of brainstem deformity and craniocervical instability. This review of literature and prospective pilot study is the first to address the potential correlation between correction of the pathological CXA and postoperative clinical outcome. The CXA is a useful sentinel to alert the radiologist and surgeon to the possibility of brainstem deformity or instability. Ten adult subjects with ventral brainstem compression, radiographically manifest as a kyphotic CXA, underwent correction of deformity (normalization of the CXA) prior to fusion and occipito-cervical stabilization. The subjects were assessed preoperatively and at one, three, six, and twelve months after surgery, using established clinical metrics: the visual analog pain scale (VAS), American Spinal InjuryAssociation Impairment Scale (ASIA), Oswestry Neck Disability Index, SF 36, and Karnofsky Index. Parametric and non-parametric statistical tests were performed to correlate clinical outcome with CXA. No major complications were observed. Two patients showed pedicle screws adjacent to but not deforming the vertebral artery on post-operative CT scan. All clinical metrics showed statistically significant improvement. Mean CXA was normalized from 135.8° to 163.7°. Correction of abnormal CXA correlated with statistically significant clinical improvement in this cohort of patients. The study supports the thesis that the CXA maybe an important metric for predicting the risk of brainstem and upper spinal cord deformation. Further study is feasible and warranted.

## Introduction

Craniocervical junction malformations and instability are not uncommon in degenerative and heritable disorders of collagen and bone [15]. Recent work by Brockmeyer and Bollo has refocused attention upon the clivo-axial angle (CXA), as an important metric in the formulation of risk of craniocervical instability, and in the determination of need for fusion and stabilization in patients with craniocervical malformations [10]. The CXA varies from 150° in flexion to 180° in extension. Van Gilder was the first to suggest that a CXA less than 150° may be associated with ventral cord compression [80, 158]. Others reported that the kyphotic CXA in traumatic, developmental, heritable hypermobility, or degenerative conditions may cause deformation of the brainstem and upper-cervical spinal cord [18, 65, 83, 99, 147] and that there may be salutary consequences to the correction of the CXA [15, 65, 81, 89]. Concurrent to the recognition of the kyphotic CXA and brainstem deformation, has been the growing understanding of mechanically induced neural injury [5, 18, 30, 55, 61, 63–68, 82, 91, 97, 153, 156, 161, 162].

This review and pilot clinical study was undertaken to address the question of whether we can reasonably establish from the literature, a posteriori, that mechanical deformation of the brainstem causes neurological deficit, and second to determine the feasibility of establishing a correlation between measured neurological performance and correction of the CXA.

## Methods and materials

### Surgical criteria

**Fig. 1:**
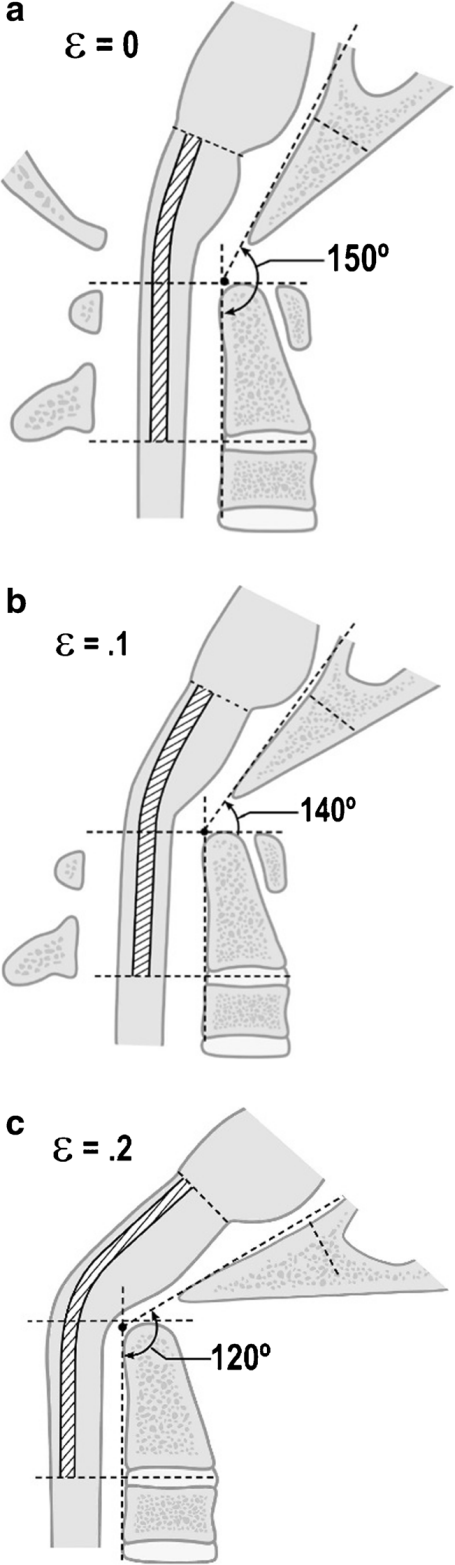
**a** Normal craniocervical junction in the neutral position. The CXA varies from 150° to 165°. There is minimal or zero deformative strain in the neutral state. **b** Normal craniocervical junction in flexion. The neuraxis stretches by approximately 10% of its total length with flexion of the craniocervical junction creating a strain ε = 0.1. **c** Pathological craniocervical junction with an abnormal CXA in flexion. Upon full flexion at the craniocervical junction, the increase in the tangent arc creates a deformative strain approaching ε = 0.2 (i.e., 20% stretch). In vivo and in vitro models demonstrate decreased or loss of neurological function with strains of 0.2

Ten adult patients were prospectively entered into the pilot study from 2003 to 2005 at Georgetown University Hospital for the following surgical criteria: (i) moderate to severe headache or suboccipital pain, (ii) bulbar symptoms constituting the cervical medullary syndrome, (iii) neurological findings of myelopathy, and (iv) CXA less than 135° (Fig. 1a). Brainstem symptoms that constitute the cervical medullary syndrome [7] are listed in Table 1. Presenting symptoms of the patient subjects are listed in Table 2. Data was deidentified, and patients were assigned nonsequential treatment numbers. With the exception of the results of the neurological exam, the data was collected by a nontreating assistant to reduce bias. Measurements by surgeons and radiologists contend with variability, and therefore, multiple methodologies for assessing interrater reliability have been established [149]; while applying such a methodology to studies of the CXA may be warranted in future, the goal of this study was not to establish reliability among radiologists, but to have a single method consistently utilized by one reader whose opinion was then utilized to direct and assess clinical outcomes.

**Table 1:** Bulbar symptoms index

The following 20 symptoms may be referable to pathology at the level of the brainstem. Please indicate "yes" or "no" whether you have any of the following symptoms on a recurring or chronic basis.
Double vision
Dizziness
Vertigo
Ringing in the ears
Speech difficulties
Difficulty swallowing
Sleep apnea
Snoring or frequent awakening
Memory loss
Choking on food
Hands turn blue in cold weather
Numbness in your arms and shoulders
Numbness in your back and legs
Get tired very easily
Unsteady walking
More clumsy than you used to be
Urinate more often (every 1–2 h)
Irritable bowel disease or gastro esophageal reflux disease
Weaker than you would expect in your arms or hand
Weaker in your legs

Five percent each positive response, 0–100%

**Table 2:** Patients and symptoms

Patient ID	Age	Sex	Presenting diagnosis and symptoms	Postoperative symptoms
G20	37	F	Basilar invagination; extremity numbness, weakness in arms (right) and legs, painful prickling from hands to scalp, blurred vision, loss of coordination, headaches, low back pain, chronic fatigue	Resolution of all symptoms, some numbness in hand and feet remained, no weakness
G13	33	F	Basilar invagination with syringomyelia; headaches, seizure-like episodes, nystagmus, increased motor tone	Resolution of all symptoms
G17	44	M	Basilar invagination; headaches, memory loss, pain, gagging, vertigo, progressive weakness, sensory loss, blurred vision, increasing bowel and urinary difficulties	Resolution of headaches, pain, vertigo, blurred vision, and sensory loss
G8	55	F	Basilar invagination; urinary frequency, incontinence, sexual difficulties, numbness, weakness, clumsiness, fatigue, memory difficulties, ringing in ears, neck stiffness, quadriparesis	Resolution of all symptoms
G2	80	F	Inflammatory thickening of transverse odontoid ligament and synovium; neck stiffness and pain, left patellar tendon hyperreflexia, left-sided dysdiadochokinesia	All symptoms resolved, some difficulty swallowing
G7	65	F	Basilar invagination with Klippel-Feil syndrome; neck pain, patchy sensory loss, absent gag reflex, balance and urinary difficulties	Normal strength and sensation, some hypoesthesia at C5, pain reduced but not absent
G3	37	M	Basilar invagination; progressive neck pain	Occasional dizziness with rapid head turning
G9	65	M	Basilar invagination; fatigue and numbness in left arm and leg, visual changes, dizziness, vertigo, GERD, headaches, urinary frequency	All symptoms resolved, some left-sided dysdiadochokinesia
G6	63	M	Basilar invagination; sleep apnea, spasticity, weakness, some sensory loss, neck pain, urinary difficulties	Normal strength and sensation, neuralgia paraesthetica on left side, resolution of all brainstem symptoms
G14	58	M	Basilar invagination; Urinary frequency, incontinence, sexual difficulties, numbness, weakness, clumsiness, fatigue, memory difficulties, ringing in ears, neck stiffness, quadriparesis	Resolution of all symptoms

### Surgical procedure

**Fig. 2:**
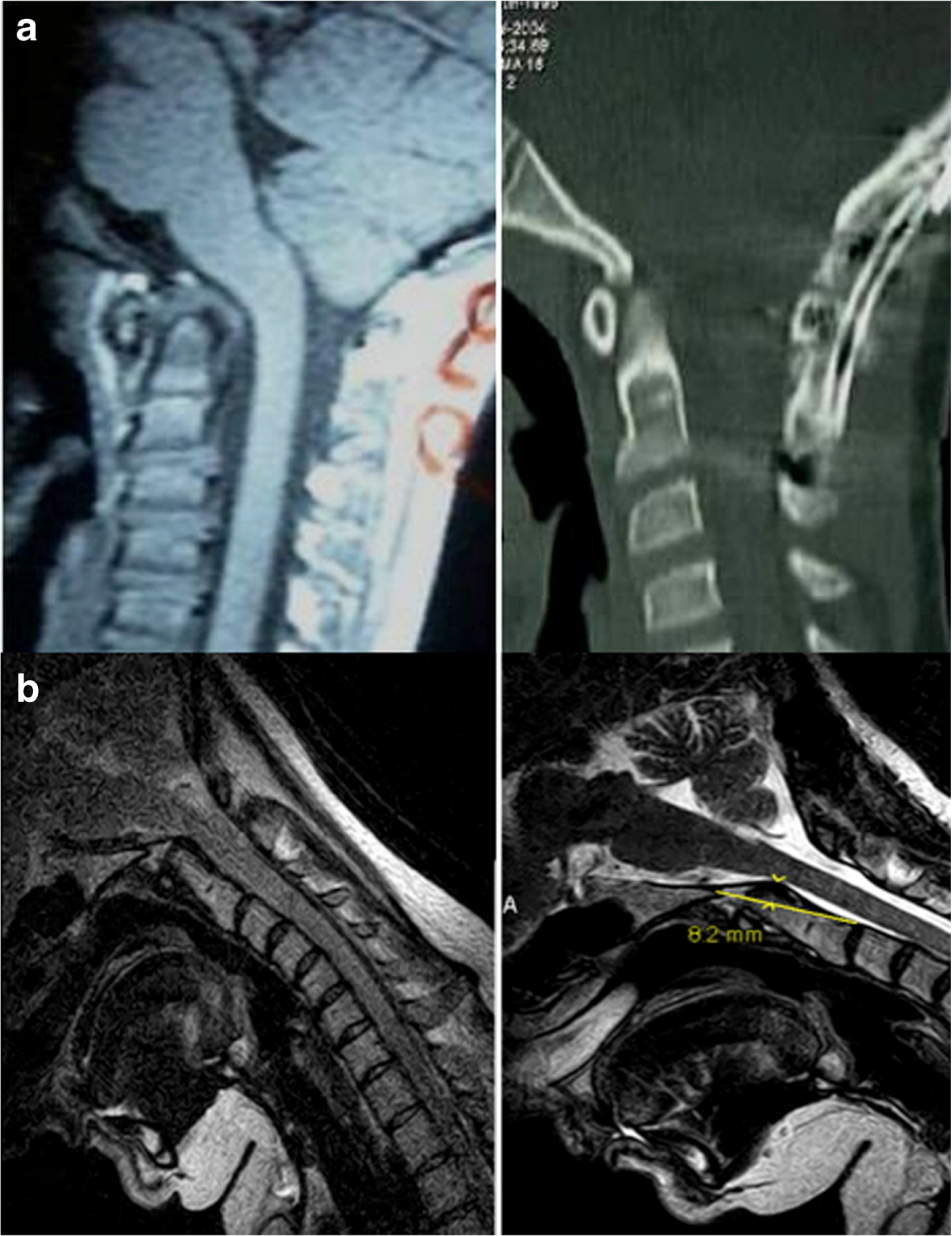
**a** Preoperative and postoperative CT of a patient showing correction of the CXA and stabilization of the craniocervical junction. **b** Preoperative and postoperative MRI in the flexed position showing the results of intraoperative correction of the CXA to straighten the neuraxis and thereby reduce the neuraxial strain

**Fig. 3:**
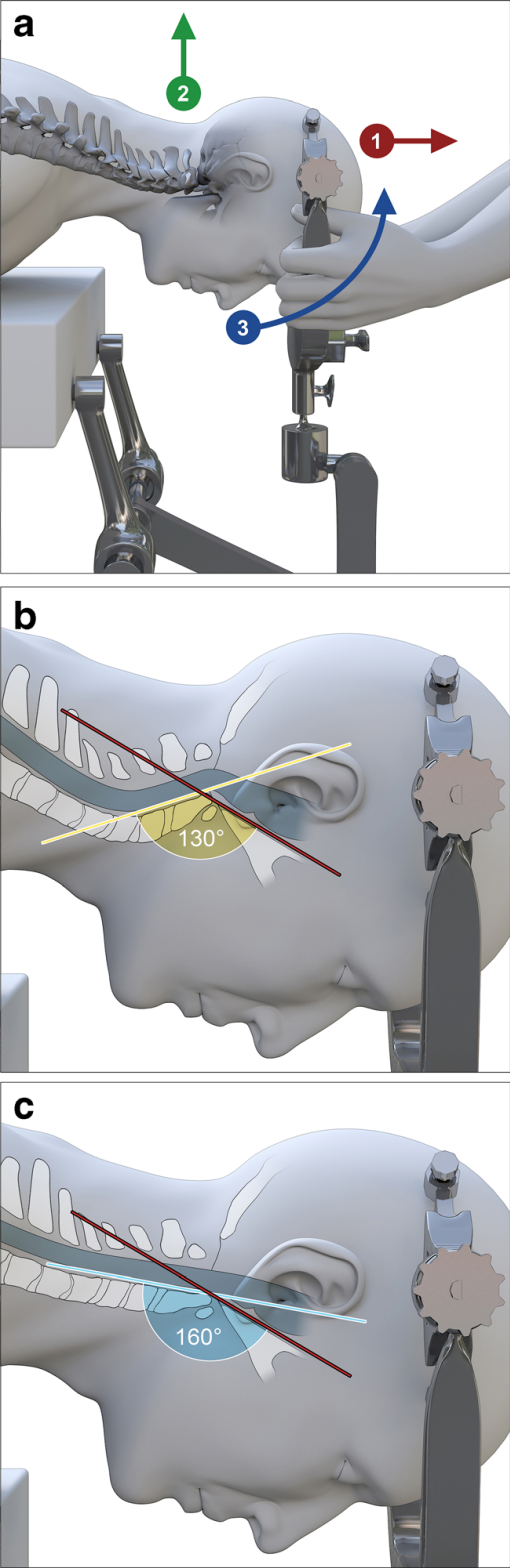
Open reduction of the kyphotic CXA. The technique described in Kim et al. (2004) is modified: the head is placed in a Mayfield head holder during exposure; during correction of the CXA, the surgeon breaks from scrub to take hold of the Mayfield head holder; another assistant releases the Mayfield clamps; correction of the CXA is then accomplished by the surgeon who places the head in slight traction (3–5 lbs), extends the head at the craniocervical junction approximately 20°, and posteriorly translates the skull by lifting, to align the basion with the odontoid process (**a**, **b**). The assistant then locks the clamps and fluoroscopic imaging is performed to measure the CXA, the position of the basion with respect to the odontoid, the “gaze” angle, and the presence of cervical lordosis (**c**). The maneuver may require two to four iterations before the final and optimal alignment is confirmed by fluoroscopy

The goal of surgery was to reduce the medullary kyphosis (the bending of the brainstem) over the odontoid, by straightening the CXA [46, 48, 52, 81, 137, 158]. After open reduction of the kyphotic CXA (that is, normalization of the CXA), subjects underwent stabilization and fusion to preserve this corrected relationship (Fig. 2a). Only the subocciput and upper two or three vertebrae were exposed. Suboccipital decompressive craniectomy was not performed. Correction of the CXA was performed by the first author, in the manner similar to that described by Kim, Rekate, Klopfenstein, and Sonntag [81]. The patient was positioned prone in a Mayfield head holder (Fig. 3a). Sensory and motor evoked potentials were monitored throughout the procedure. The reduction was accomplished in one to four iterations, under fluoroscopic guidance, by applying traction to the cranium, posterior translation, and then extension to establish a more normal CXA, with the basion above the midpoint odontoid process (Fig. 3c) [58].

To accomplish the stabilization, a titanium plate (Altius™, Biomet, Parsippany, NJ) was contoured to the occiput and screwed to the skull. This plate was connected by rods to screws placed in the C1 lateral mass, the C2 pedicles, and where necessary, to screws in the C3 lateral masses. The bone surfaces were decorticated, and the fusion completed with two rib autografts, contoured from the suboccipital bone to the upper cervical vertebrae, and augmented with demineralized bone matrix [138]. Both wounds were then closed over drains. The patients were usually mobilized 1 day after surgery and kept in a neck brace (Miami J™, or equivalent) for 6 weeks.

### Clinical metrics

Subjects were evaluated preoperatively and postoperatively at 1, 3, 6, and 12 months for quality of life (Short Form 36 (SF-36)), neurological function (American Spinal Injury Association Impairment scale (ASIA)), pain (Visual Analog Scale (VAS)), neck disability and pain (Oswestry Neck Disability Index), overall function (Karnofsky Index), and presence of bulbar symptoms (the Brainstem Disability Index—20 questions relating to bulbar symptoms, see Table 2). The bulbar (brainstem) symptoms (cervical medullary symptoms) score is a numeric representation of the number of bulbar symptoms with which the subject presented before and after surgery (Table 1) [7]. The CXA were measured independently by neuroradiologists. Test/retest reliability of the CXA measurement is not available.

## Results

Average surgical duration was 3.5 h. All subjects were discharged within 3 days of surgery. Sensory evoked potentials were monitored throughout each surgery and revealed no decrement in amplitude or increased latency.

### Complications

Postoperative CT showed two C2 pedicle screws entering slightly the vertebral artery foramen, but no compression of the vertebral arteries, and there were no symptoms or signs of vertebral artery compression. One subject lost approximately 500 ml of blood intraoperatively, but did not require transfusion. One patient reported worsened swallowing after surgery. There were no complications related to harvest of autologous rib for fusion, although several patients reported discomfort over the rib harvest site, which is captured in the VAS, overall pain metric.

### Clivo-axial angle

The preoperative mean CXA was 135.8° in the neutral view (range 131°–140°). While the pathological threshold for the CXA in this report was considered 135°, one subject who had been followed for 9 months was admitted to the study with a CXA of 140°, because of the progressive pain, the compelling neurological findings, and the response to the neck brace. After open reduction, the mean CXA increased to 163.7° at the 12-month follow-up (range 150°–176°) in the fused position (Fig. 3a, b).

### Neurological signs and symptoms

Common symptoms included headache or neck pain, memory loss, hypoesthesias or paresthesias, clumsiness with frequent falls, imbalanced gait, and weakness in the upper or lower extremities. Several subjects reported reflux gastritis or irritable bowel syndrome, sleep apnea (or history of unrestful sleep and frequent awakening), vestibular, auditory and visual disturbances, and bowel and bladder dysfunction. One patient reported sexual difficulties and another spasticity (Table 2).

Preoperative neurological findings included weakness, especially hands and limbs, poor posture; dysdiadochokinesia; sensory changes; hyperreflexia; and scoliosis. The sensory changes most prominently included hypoesthesia to pinprick, but never painful or unpleasant, and was frequently ignored or unrecognized by the patient until examination. The gag reflex was decreased or absent in all subjects, though usually not associated with dysphagia.

Postoperatively, every subject reported substantial improvement in most symptoms. Improvement continued over the 1-year follow-up period. Those symptoms that failed to resolve are noted in Table 2.

### Clinical metrics

A summary of clinical data is presented in Table 3. The preoperative CXA and clinical metrics were compared with those at 12-month follow-up. The normalized SF-36 physical component scores increased from a mean of 38.09 to a mean of 50.98; Mental Component scores improved from a mean of 45.68 to 56.31 (*p* = 0.0008). Mean pain, measured by the VAS decreased from 5.6 to 1.1 (*p* = 0.0009). Oswestry Neck Disability Index scores decreased from a mean of 38.75 to a mean of 10.89 (*p* = 0.006). Mean ASIA score improved from 296.4 to 314.8 (*p* = 0.004). Mean Karnofsky score increased from 80 to 97 (0.0003). The mean number of brainstem symptoms per patient decreased from 10.3 to 2.3 symptoms. Nonparametric Wilcoxon signed-rank tests were statistically significant (*p* < 0.02 for all tests). The patients’ responses to each question in the list of bulbar symptoms (Brainstem Disability Index) preoperatively and at 12-month follow-up are listed in Table 4.

**Table 3:** Mean clinical metrics

	Preoperative mean	12-Month follow-up mean	*p* value from nonparametric test
SF-36 Physical Component	38.09	50.98	0.010
SF-36 Mental Component	45.68	56.31	0.006
Karnofsky Scale	80	97	0.008
Visual-Analog Pain Scale	5.6	1.1	0.007
Oswestry Neck Disability Index	38.75	10.89	0.016
ASIA Scale	296.4	314.8	0.014
Number of bulbar symptoms	10.3	2.3	0.009

**Table 4:** Bulbar symptoms before and after surgery

Symptom	Number of patients affected before surgery	Number of patients affected at 12-month follow-up
Double vision	5	0
Dizziness	6	1
Vertigo	3	0
Ringing in the ears	6	3
Difficulty swallowing	3	1
Sleep apnea	5	0
Snoring	6	4
Memory loss	5	1
Choking on food	2	1
Hands turn blue in cold weather	3	0
Numbness in arms and shoulders	6	1
Numbness in back and legs	4	2
Get tired easily	8	2
Unsteady walking	7	1
Clumsiness	9	0
Urinary frequency	7	2
Irritable bowel or GERD	4	1
Sexual difficulty	3	1
Weakness in arms and hands	8	0
Weakness in legs	3	2

## Discussion

There is growing recognition that the CXA is an important metric to assess the risk of brainstem deformity and the potential need for reduction and stabilization of the craniocervical junction [7, 10, 15, 21, 65, 81, 89, 94, 114, 158]. The kyphotic CXA is an anatomic deformity, introducing a medullary kink and concomitant increase in biomechanical stress of the brainstem or upper spinal cord, and presenting clinically with pain, the cervical medullary syndrome, and usually neurological deficits. This study specifically addresses the question as to whether there is a correlation between the clinical findings of the cervical medullary syndrome and the kyphotic CXA (<135°), and whether correction of the CXA, that is reduction of kyphosis, correlates with clinical improvement.

### Clinical outcomes

With the exception of the Brainstem Disability Scale, all metrics in this study are validated and widely used. The SF-36 instrument measures general health, vitality, physical functioning, bodily pain, social functioning, and mental health; its validity is established [16, 43, 44]. The ASIA scale is a useful metric for registering subtle changes in sensory and motor function. The Karnofsky index, designed as a functional index for cancer patients, has been generalized as an instrument for functional assessment in a broader category of patients [34]. The Brainstem Disability Symptom Index, used elsewhere [65], is simply a collation of brainstem symptoms, approximating the cervical medullary syndrome, arising from brainstem compression [7, 31, 48, 49, 52, 57, 81, 105, 106, 108, 111, 117, 137]. A score of 100 represents the presence of all 20 symptoms and significant disability (Table 1). This cohort of subjects, who presented with moderately severe pain, brainstem symptoms comprising the cervical medullary syndrome, myelopathy and a kyphotic CXA, underwent open reduction to normalize the CXA, and stabilization. Postoperatively, clinical improvement was statistically significant for each metric of pain (VAS), Oswestry Neck Disability Index, sensorimotor function (ASIA scale), overall performance (Karnofsky Performance Index), quality of life, mental and physical (SF-36), and brainstem symptoms (Brainstem Disability Index), and these improvements correlated with normalization of the CXA. With the exception of the neurological examination, a research assistant collected the data; the subjects were, therefore, not subject to the influence of the surgeon in the subject interview.

These clinical results are consonant with those of others, [47, 52, 81, 85, 90, 108, 136, 139] including Kim et al. who attributed the improvement in subjects with abnormal CXA to reduction and stabilization [81], and Goel who reported that restoration of craniospinal alignment resulted in “remarkable and sustained clinical recovery” [46, 48]. These results compare favorably with series in which the CXA and the potential for craniocervical stability were not taken into account [3, 57, 89].

Among the brainstem findings, absent gag reflex, vocal cord dysfunction, and facial sensory loss of pinprick were the most common findings, posited to result from deformation of the nucleus ambiguus and the trigeminal nucleus, respectively [32]. Respiratory and gastrointestinal disorders were highly represented in this series, as in others [2, 13, 14, 25, 39, 42, 51, 63, 65, 66, 96, 111, 122, 123, 131, 133, 143].

### The clivo-axial angle

The CXA is variably defined as the clivus vertebral angle [158], the clivus canal angle [15], the clivus-cervical angle, and the clivus-axial angle. Botelho describes the CXA as the angle between the line extending from the top of the dorsum sellae to the basion, and the line between the infero-dorsal to the most superodorsal part of the dens. Others have used a line drawn through the mid potion of the odontoid [10]. With the goal of standardizing terminology and methodology, the subject of the CXA was recently addressed in multidisciplinary consensus statement that describes the CXA as the angle between the clivus line and the posterior axial line [7]. The clivus line is drawn along the lower third of the clivus, from the spheno-occipital synchondrosis to the basion; in the case of basilar invagination, it is drawn from the spheno-occipital synchondrosis to the top of the odontoid process. The posterior axial line is differentiated to reflect either the bone contour of the axis on CT, the so-called *bone CXA*, or the ligamentous margin of the odontoid—the *soft tissue CXA*. The soft tissue CXA, necessarily including thickening of the posterior ligament due to pannus, may be more pertinent in identifying possible ventral brainstem compression, and is therefore more representative of the pathology. [7].

The CXA has a normal range of 145° to 160° in the neutral position. Nagashima and Kubota directly measured the normal CXA as 158.2° ± 9.8° in normal adults; women had an increased range of motion compared to men. Flexion of the neck decreases the CXA by 9° to 11° and extension increases CXA by the same [114, 159]. In a series of 41 patients with atlanto-axial subluxation due to rheumatoid arthritis, the average preoperative CXA was 153° [114]. Botelho and Ferreira performed detailed craniometrics on 106 individuals: 33 as controls, 48 with Chiari I malformation, and 25 with basilar invagination. The control group had a mean CXA of 148° (range 129°–175°, std.dev. 10°), the Chiari group had a mean CXA of 150° (range 123°–180°, std.dev. 12°), and those with basilar invagination had a mean CXA of 120° (range 79°–145°). Not unexpectedly, in the rheumatoid arthritis population, where basilar invagination and instability is common, the CXA ranges from 135° on full flexion to 175° on extension [21]. In our surgical cohort, the CXA increased from an average 135° preoperatively to 162° postoperatively.

### The importance of the abnormal clivo-axial angle

Van Gilder reported that a clivus vertebral angle (CXA) of less than 150° was associated with neurological changes [121, 130]. Nagashima and Kubota reported that a CXA less than 130° may produce ventral brainstem compression, and should “be corrected to a greater angle during the fusion stabilization” [114]. Others have cited the importance of “medullary kinking” due to basilar invagination, kyphotic angulation of the brainstem [61, 63, 81, 105, 111, 140, 154] retroflexed odontoid process [111] and nontraditional basilar invagination [52, 81, 111, 137]. Kubota reported on a series of Chiari I malformation subjects, in whom the syringomyelia failed to resolve in those patients with a kyphotic clivo-axial angle (<130°) [89].

Flexion of the craniocervical junction causes brainstem lengthening, normally by 10% (Fig. 1a–c) [18]. Sawin and Menezes described the “fulcrum effect in basilar invagination, by which traction is applied to the caudal brainstem and rostral cervical spinal cord, producing prominent bulbar dysfunction and myelopathy” [137]. They recognized progression of disability in many patients following suboccipital decompression for Chiari I, attributing the observed brainstem findings to this fulcrum effect [108].

Morishita et al. reported that measurement of the cervico-medullary angle helps to identify instability of the occipito-atlantoaxial junction and that angles less than 135° indicated atlanto-axial impaction and myelopathy [113]. Botelho reported that normalization of the CXA in a patient with basilar invagination reduced the ventral brainstem compression: “the effect of ventral brainstem compression was clearly observed in this patient because numbness in the hands was readily ameliorated after traction” [12].

One large series reported “kinking of the medulla” (kyphotic CXA) in 140 of 364 of Chiari patients [111]; these were recognized as a form of “functional cranial settling” [110] in one series, and cranial settling or basilar invagination occurred in 25–30% of cases of Chiari malformation [26]. Kim et al. described “nontraditional basilar invagination” as the underlying cause of recurrence of pain and disabling symptoms in Chiari I patients after conventional suboccipital decompression, and reported substantial neurological improvement after intraoperatively correcting the CXA from an average of 127° to 147° [81]. Functional cranial settling, such as occurs with connective tissue disorders [110], may invite inordinate aggregate translation and flexion and subsequent basilar invagination with compression of the spinal cord or medulla [53, 54, 61, 64, 65, 116, 165].

Horizontal translation of the cranium and horizontal tilting of the odontoid may also occur. Translation occurs between the occiput and atlas [40, 157]. List in 1941 recognized that anterolisthesis of the cranium on the spine resulted in disabling neurobehavioral disorders [94]. The “retroflexed odontoid” may deform the brainstem, especially in flexion, a fact which underscores the importance of dynamic images [50, 84, 110, 111].

### The relationship between the basal angle and the clivo-axial angle

Platybasia is defined by the *basal angle*, formed by a line extending across the anterior fossa from the nasion to the tip of the dorsum sella, and a second connecting line drawn along the posterior margin of the clivus. In normal adults, the basal angle is 116° ± 6°, and in children 114 ± 5° [69]. Therefore, assuming verticality of the axis and simple geometric considerations, the normal CXA computes to 154° ± 6°. Naturally then, as the basal angle increases (becomes more flattened), the CXA becomes more kyphotic and pathological [15, 114]. Pang notes that platybasia must “necessarily narrow the clivus canal angle,” and that the short clivus and raised basion likely “underlie most forms of basilar invagination, especially those with a reflex dens” [120]. Nohria and Oakes noted the association of abnormal CXA in association with platybasia [117]. Goel noted an abnormal basal angle in 124 of 190 patients with basilar invagination, necessitating that in the majority of his cases, there was also an abnormal CXA [47].

Platybasia is often associated with encephalomyelopathy from medullary kink [24, 47, 49, 103, 129, 140] from degenerative conditions such as rheumatoid arthritis and other causes of hypertrophy of the odontoid [23, 29, 63, 66] in inherited conditions such as Ehlers-Danlos syndrome [110], achondroplasia [59, 79, 133, 151, 163], osteogenesis imperfecta [88, 144], Hurler’s syndrome, and from acquired bone-softening conditions such as rickets, hyperparathyroidism, spondyloepiphyseal dysplasia, acro-osteolysis, osteomalacia, achondromalacia, renal osteodystrophy, and Paget’s disease, in which abnormal bone remodeling causes bone weakening and subsequent platybasia and basilar invagination [19, 20, 69, 70, 104, 112]. Platybasia may therefore signal the presence of a pathological CXA.

### Dynamic MRI

Basilar invagination that is not present on neutral views may become evident with dynamic imaging *during flexion* of the craniocervical junction [65, 85, 110]. Odontoid instability and connective tissue disorders, such as rheumatoid arthritis, Down’s, Morquio, Marfan, and Ehlers Danlos syndromes, may be associated with pathological instability. The importance of dynamic flexion–extension imaging to assess instability would appear reasonable: craniocervical instability was present in 9 of 29 patients with Down’s syndrome patients investigated by Spitzer [148]. Menezes reported upon 100 children with Down’s syndrome, of whom 54 presented with symptoms referable to the craniocervical junction; dynamic imaging revealed 24 subjects with craniocervical instability and 34 with C1–2 instability, including 23 with rotary instability; 25 subjects underwent occipito-cervical fusion and stabilization, with resolution or near resolution of neurological symptoms in almost all subjects [107]. Dynamic films demonstrate not only the degree of instability but also the potential for reduction and the anatomic proximity of the bone and ligament to the nervous system [26].

### Criteria for surgery

The subjects of this cohort were referred from neurologists for headache and neck pain, bulbar symptoms, myelopathy, and radiological findings of basilar invagination or ventral brainstem compression. While small Chiari I malformations (less than 7 mm) were present in this study cohort, patients with larger Chiari malformations—which clearly required decompression—were not included in this study. Therefore, no suboccipital decompressions or C1 laminectomies were performed in this cohort. It is recommended, however, that clinically significant Chiari malformation with craniocervical instability or basilar invagination complex Chiari malformations—which is to say, “*the complex Chiari*”—would be considered for suboccipital decompression, reduction, fusion, and stabilization [11, 47, 60, 81, 89]. Patients with severe basilar invagination, such as from rheumatoid arthritis, osteogenesis imperfecta, achondroplasia, or Paget’s disease, though not represented in this study, would usually undergo open traction-reduction, posterior stabilization, fusion, and with persistence of clinically significant basilar invagination, would undergo ventral decompression [23, 57, 112, 137].

### Reduction

In this study, the CXA was normalized, and that new relationship stabilized [4, 74, 119, 123, 130] in the same manner as others have recommended for progressive neurological deficits [33, 47, 56, 86, 145, 159] and where dynamic imaging revealed pathological motion at the craniocervical junction [23, 29, 36, 109].

The recognition that reduction can be accomplished with traction and extension of the craniocervical junction is well established [12, 46–49, 65, 66, 81, 85, 89, 110, 135, 137]. Nagashima and Kubota established that the acute CXA could be “corrected with… staged traction intraoperatively, in the majority of cases” [114]. This has been the experience of others [12, 46, 81, 165]. A more complex surgical solution is required in those cases in which there appears to be an irreducible atlanto axial dislocation, where cervical laminectomy may be required at C1 or C2 [135] or severe basilar impression in osteogenesis imperfecta, rheumatoid arthritis, or Paget’s disease—in whom traction reduction for several days prior to surgery should be considered [100, 137, 145] or in whom ventral decompression is required.

Where more severe basilar invagination exists and further decompression is indicated, a transoral or transnasal endoscopic decompression of the odontoid may be indicated. Microsurgical transoral decompression is encumbered by dysphagia, often severe lingual edema and dysphonia. The more recently developed endoscopic techniques, however, provide a substantially equal field of view without dysphonia and oral edema, and the patient can be safely extubated after surgery. While the anterior decompression can usually be accomplished safely as an initial procedure, the authors propose performing the posterior fusion stabilization first, and then reassessing the patient to ensure the necessity of the anterior decompression. Performing the posterior stabilization initially does not result in limitation of anterior access to the nasal or oral pharynx, nor restrict the field of view from the anterior approach. In the case of significant instability, moreover, the posterior stabilization may increase the safety during positioning and performance of the anterior approach [128, 132].

While a more acute form of injury, such as trauma, may dictate urgent decompression and stabilization, the vast majority of cases of brainstem kinking and compression occur in the realm of chronic trauma, wherein the ultimate pathophysiological substrate of injury devolves upon the repetitive, mechanical deformative stresses imposed upon the neuraxis [22, 62, 63, 73]. Treatment is dictated, therefore, by the least morbid means of establishing reduction of deformity and stabilization. This is primarily, and most expeditiously, accomplished posteriorly; however, when anterior deformity remains, an anterior decompression should be considered.

**Fig. 4:**
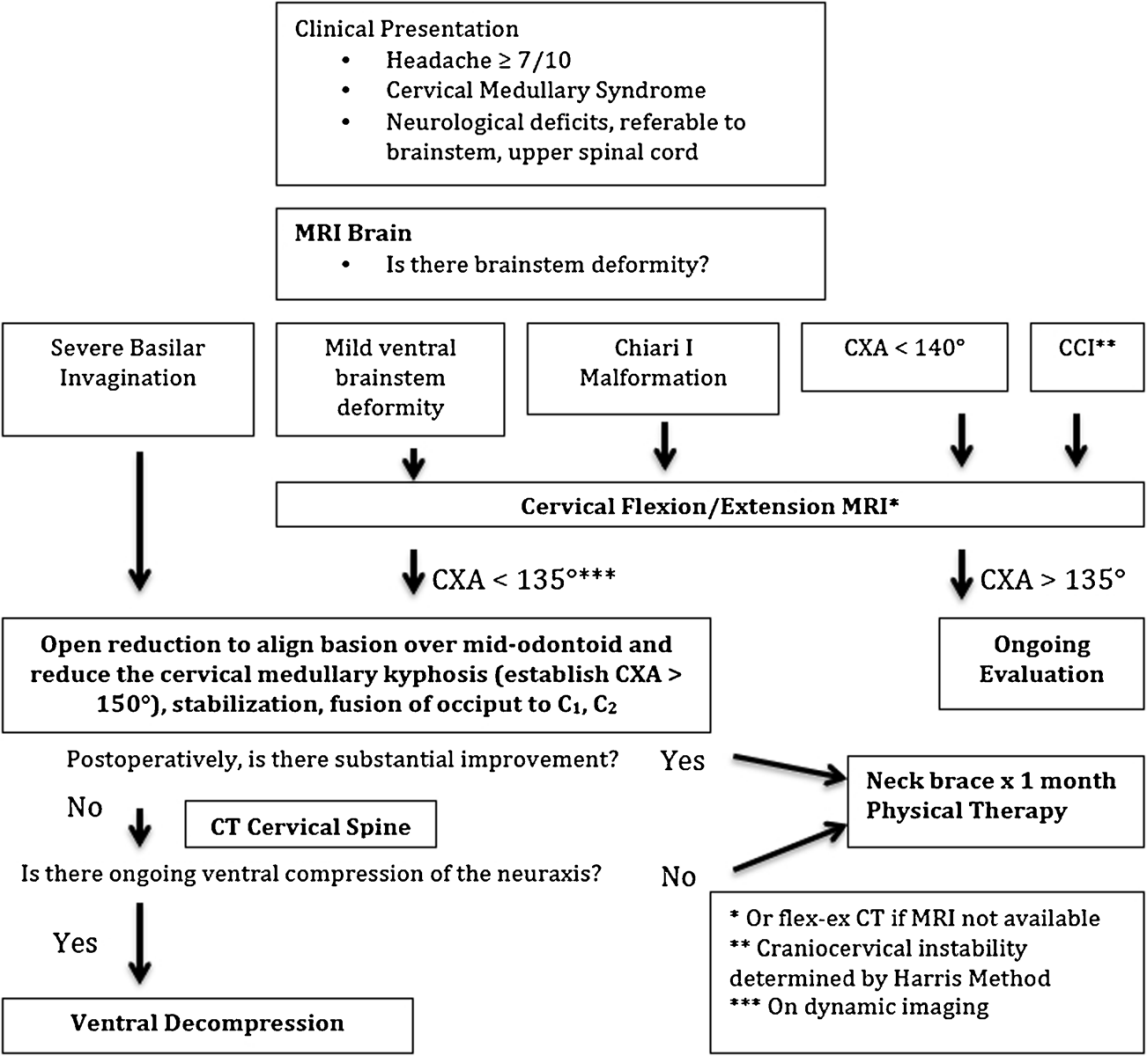
Algorithm for correction of kyphotic CXA

The authors propose an intuitive algorithm, for subjects disabled from headache and neurological findings referable to the lower brainstem or upper spinal cord, which in aggregate are called the *cervical medullary syndrome* [7]. If the MRI shows severe basilar invagination, then the patient should undergo a posterior reduction (to reduce the kyphotic CXA) and fusion/stabilization. If clinically significant basilar invagination persists postoperatively, then a transoral or transnasal odontoidectomy is indicated. On the other hand, the presence of mild ventral brainstem deformity, Chiari I malformation, kyphotic CXA (<140°) or neurologic findings suggestive of craniocervical instability, warrants further evaluation with dynamic flexion/extension imaging, preferably MRI. The demonstration of a pathological CXA (<135° on flexion), craniocervical instability, or ventral brainstem compression warrant a nonoperative trial (neck brace and physical therapy), and then consideration for occiptocervical reduction, fusion, and stabilization. If clinically significant basilar invagination persists, then a ventral decompression should be considered. (Fig. 4)

### Complications

No major complications were observed in this cohort. In two subjects, postoperative CT showed pedicle screws adjacent to, but not compressing, the vertebral artery at C2. In each case, the subsequent MRA was normal. However, the authors do not want to minimize the real risk associated with injury to the vertebral artery on placement of the C2 pedicle screws [37, 98, 115]. While there were no complications related to harvest of autologous rib for fusion, the majority of subjects report prolonged discomfort over the rib harvest site; furthermore, rib harvest can cause a mild instability in the thoracic spine, and possibly scoliosis. Though autologous rib has in the past been considered the gold standard for occipito-cervical fusion [138], the authors now avoid rib harvest pain by using allograft augmented with aspirated bone marrow to accomplish the fusion.

Kim et al. reported a 36% complication rate, primarily comprised of minor complications, but one patient in whom hyperostosis necessitated posterior decompression [81]. Kumar reported two deaths due to spinal cord injury, sustained when the patient was being turned prone after transoral decompression [90].

The authors are unaware of delayed complications of occipito-cervical fusion in this cohort, where fusion was limited to the upper cervical vertebrae. Despite the loss of 35° of neck rotation to each side and 21° of flexion and extension between the occiput and cervical spine [159] only one subject reported concern about the decreased range of motion. This is probably due to compensation at lower cervical levels [85], compensatory torso rotation [135], and remodeling of vertebrae [154]. Excessive reduction at the atlanto-axial level may result in the development of kyphosis or swan neck deformity at subaxial levels [118, 154, 164], but Iizuka [73] demonstrated that the CXA appears to be independent from surgical reduction of the atlanto-axial angle.

Nockels’ and Shaffrey’s series of occipitocervical fusion/stabilization for craniocervical anomaly reported no complications and 87% improvement of myelopathy. They concluded that “rigid internal fixation of the occipito-cervical complex is safe and effective for spine surgeons familiar with the occipital bone anatomy and lateral mass fixation” [116]. Overall, published data for craniospinal fusion stabilization shows that the morbidity is comparable to lumbar discectomy [121].

### Pathophysiology

Scoville and Sherman first opined that angulation of the brainstem in basilar invagination caused neurologic signs and disability [140]. Others have agreed [8, 17, 18, 23, 28, 45, 52, 61–63, 65, 71, 72, 77, 81, 89, 94, 99, 108, 111, 114, 114, 114, 114, 114]. Menezes noted clinical improvement after “relief of brainstem angulation” [108]. While the preoperative neurological deficits have been attributed by some to impaired blood supply, the rapid neurological improvement that follows correction of neuraxial deformity suggests that preoperative neurological changes were *not* due to long-standing ischemia [61, 63, 135]. The rapid clinical improvement is more likely due to elimination of the craniocervical instability and reduction of deformity, as measured by correction of the CXA, and the observed recoverability in these chronic injuries is consistent with the observation in experimental models that axons subjected to strain recover rapidly, both anatomically and functionally [17, 18, 93, 126, 141, 153].

The predominant substrate for deformity-induced injury is the axon. The deformation, or stretching, of the axons occur with flexion of the neck [17, 61, 131, 146]. A 20% stretch (strain ε = 0.2) renders the giant squid axon nonconductive [41] and results in the development of axon retraction balls in the murine optic nerve [134]. Electron micrographs show clumping, loss of microtubules and neurofilaments, loss of axon transport and accumulations of axoplasmic material identified as the retraction ball [61, 63, 75, 76, 100–102] analogous to diffuse axonal injury (DAI) in the brain [125, 127]. Axon retraction bulbs are the histological substrate of stretch injury in basilar invagination [22, 61, 125, 127] and injury to the cortico-spinal tracts of the brainstem in infants with “shaken baby syndrome” [45].

The addition of compression (“out-of-plane” loading), due to cerebellar ectopia, odontoid pannus, or retroflexion, significantly increases the overall deformative mechanical stress (von Mises stress, which is the aggregate of linear strain and out of plane loading) [9, 35, 111].

The importance of stretch-related myelopathy is supported in clinical [6, 38, 64, 66, 124], experimental [83, 87, 125, 127, 131, 152], and biomechanical literature [17, 61, 72, 92, 99, 155]. The degree of injury appears to be related to the peak strain of the tissue and the rate of deformation (the loading rate) [8, 76, 142]. Even mild stretch can induce progressive neurofilament alteration and delayed axotomy [27].

Electrophysiologically, axon deformation can also result in myelin or membrane injury with decreased amplitude and increased latency [142]. Strain acts upon the Na^+^ channel mechanoreceptors to increase Na^+^ influx, reversing cation exchange pumps and depolarizing voltage-gated Ca^++^ channels and causing the pathological influx of Ca^++^ [27, 160]. Sublethally damaged neurons may undergo upregulation of N-methyl D-aspartate receptors, resulting in heightened vulnerability to subsequent challenges of reactive oxygen species and peroxynitrites, and concomitant mitochondrial dysfunction and DNA fragmentation [1]. Early calpain activation may contribute to progressive intra-axonal structural damage after stretch injury [134] or apoptosis of neurons and oligodendrocytes [1, 61, 78, 93, 95].

## Conclusions

It is reasonably established from the literature, a posteriori, that mechanical deformation of the brainstem may cause neurological deficit. The CXA appears to be a useful metric to assess potential risk of pathological deformity of the brainstem.

It is clinically feasible to correlate measured neurological performance and correction of the CXA. This prospective pilot study supports the thesis that open-reduction of cranio-spinal deformity (correction of the abnormal CXA) and stabilization yields statistically significant improvement in neurological deficit, pain, quality of life, and function, in selected subjects. However, this is a correlation only and further, appropriately powered studies are needed to demonstrate causality.

The clinical results are concordant with others and support the growing body of neurobiological evidence and mathematical modeling that deformity induces neural pathology, and correction of deformity—in this case, the normalization of the CXA—reduces the neuropathology and its protean manifestations.
